# Biomarker Discovery for Hepatocellular Carcinoma in Patients with Liver Cirrhosis Using Untargeted Metabolomics and Lipidomics Studies

**DOI:** 10.3390/metabo13101047

**Published:** 2023-10-02

**Authors:** Md Mamunur Rashid, Rency S. Varghese, Yuansong Ding, Habtom W. Ressom

**Affiliations:** Lombardi Comprehensive Cancer Center, Department of Oncology, Georgetown University Medical Center, Georgetown University, Washington, DC 20057, USA; mr1785@georgetown.edu (M.M.R.); rsv4@georgetown.edu (R.S.V.); yd239@georgetown.edu (Y.D.)

**Keywords:** liver cancer, biomarkers, metabolomic profiling, lipidomic profiling, LC-MS/MS

## Abstract

Hepatocellular carcinoma (HCC), the most prevalent form of liver cancer, is the third leading cause of mortality globally. Patients with HCC have a poor prognosis due to the fact that the emergence of symptoms typically occurs at a late stage of the disease. In addition, conventional biomarkers perform suboptimally when identifying HCC in its early stages, heightening the need for the identification of new and more effective biomarkers. Using metabolomics and lipidomics approaches, this study aims to identify serum biomarkers for identification of HCC in patients with liver cirrhosis (LC). Serum samples from 20 HCC cases and 20 patients with LC were analyzed using ultra-high-performance liquid chromatography-Q Exactive mass spectrometry (UHPLC-Q-Exactive-MS). Metabolites and lipids that are significantly altered between HCC cases and patients with LC were identified. These include organic acids, amino acids, TCA cycle intermediates, fatty acids, bile acids, glycerophospholipids, sphingolipids, and glycerolipids. The most significant variability was observed in the concentrations of bile acids, fatty acids, and glycerophospholipids. In the context of HCC cases, there was a notable increase in the levels of phosphatidylethanolamine and triglycerides, but the levels of fatty acids and phosphatidylcholine exhibited a substantial decrease. In addition, it was observed that all of the identified metabolites exhibited a superior area under the receiver operating characteristic (ROC) curve in comparison to alpha-fetoprotein (AFP). The pathway analysis of these metabolites revealed fatty acid, lipid, and energy metabolism as the most impacted pathways. Putative biomarkers identified in this study will be validated in future studies via targeted quantification.

## 1. Introduction

Hepatocellular carcinoma (HCC) is the most common form of liver cancer and ranks third on the list of primary causes of death due to cancer globally [1,2]. By 2025, it is expected that the annual incidence of liver cancer will climb to over a million cases, making HCC one of the leading causes of cancer-related mortality in several developed nations, including the United States [3]. Patients with LC have an increased risk of developing HCC due to the fact that ~90% of all HCCs are the result of long-standing cirrhosis [4]. Therefore, differentiating HCC from LC, particularly in the early stages, will be critical in the clinical decision-making.

Early diagnosis and timely intervention are crucial for improving the overall survival rates of HCC patients. Patients with HCC are often diagnosed at an advanced stage since the early stages of the disease do not produce any evident symptoms. The most used serologic marker for HCC diagnosis is alpha-fetoprotein (AFP). However, its diagnostic value has been frequently criticized because AFP is increased in only 20% of early-stage HCC patients having sensitivity, and predictive values to detect HCC range from 20 to 50%. In addition, patients with cirrhosis or hepatitis may have AFP levels that are outside the normal range [5,6]. Similarly, conventional imaging-based diagnostic approaches exhibit reduced sensitivity throughout the initial phases, resulting in diagnostic delays and poor patient outcomes [7]. Therefore, there is an urgent need for the discovery and development of sensitive and specific biomarkers that can facilitate early detection, accurate risk stratification, and personalized therapeutic strategies.

High-throughput omics technologies have provided new avenues for studying cancer, allowing researchers to better understand the intricate molecular patterns that are linked to the onset and progression of the diseases. Among these technologies, metabolomics and lipidomics offer the ability to undertake in-depth analyses of small molecule metabolites and lipid species, respectively, and thus provide insight into metabolic abnormalities in a variety of diseases, such as cancer [8,9]. Metabolomics is a rapidly growing discipline involving the systematic analyses of metabolites or small molecules in biological samples. On the other hand, lipidomics, a subset of metabolomics, primarily facilitates the evaluation of different lipid species in intricate biological samples. The metabolites and lipids in a sample reflect the underlying biological processes, making them a valuable source of information for disease diagnosis and prognosis [10].

Various omics platforms have been employed to discover potential biomarkers that can effectively detect HCC at its early stage [11]. Among these, metabolomics and lipidomics approaches were employed to analyze various categories of biological samples using manifold liquid/gas chromatography–mass spectrometry and nuclear magnetic resonance (NMR) systems. Multiple research groups have reported various classes of metabolites, including amino acids, organic acids, bile acids, fatty acids, and lipids, as potential biomarkers for the diagnosis of HCC [12,13,14,15]. However, the efficacy of the identified biomarkers in distinguishing early HCC from LC remains to be evaluated, which is the most difficult aspect of HCC diagnosis.

In the present study, metabolomics and lipidomics approaches were applied to discover HCC biomarkers by comparing with LC controls using a UHPLC-Q-Exactive-MS system.

## 2. Materials and Methods

### 2.1. Chemicals and Reagents

Internal standards (ISs) including debrisoquine sulfate, 4-nitrobenzoic acid and arachidonic acid-d8, and PC(16:0/18:1)-d31 were purchased from Sigma-Aldrich (Milwaukee, WI, USA) and Avanti Polar Lipids (Alabaster, AL, USA), respectively. Ammonium acetate was purchased from MP Biomedicals (Solon, OH, USA). Solvents for mobile phase preparation and sample extraction, including water, methanol, acetonitrile, isopropanol, and chloroform, were obtained from either Fisher Scientific (Fair Lawn, NJ, USA) or Honeywell Chemicals (Muskegon, MI, USA) and were HPLC grade.

### 2.2. Study Cohort and Sample Collection

**Figure 1:**
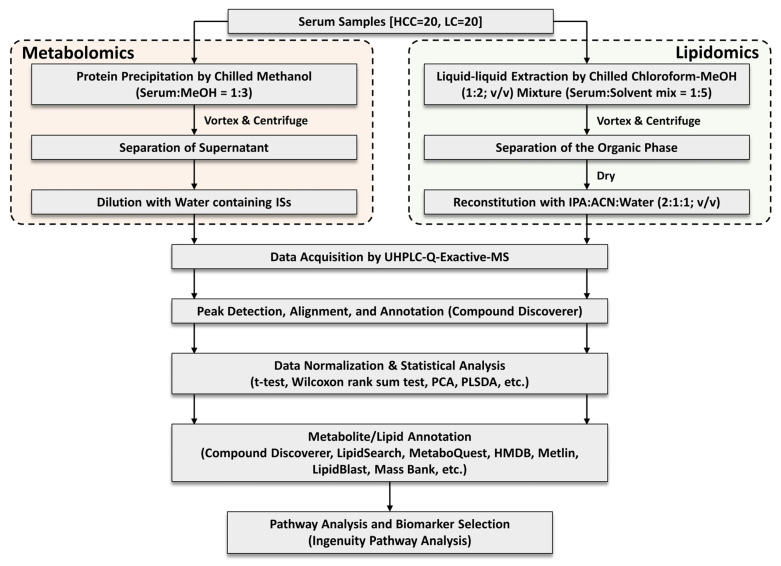
Study workflow of our LC-MS-based untargeted metabolomics and lipidomics analysis for HCC biomarker discovery. MeOH: Methanol, IPA: Isopropanol, ACN: Acetonitrile.

Serum samples from 40 adult patients recruited at MedStar Georgetown University Hospital through a protocol approved by the Georgetown IRB were analyzed in this study. All subjects provided informed consent and HIPAA authorization forms. Table 1 provides the characteristics of the 20 HCC cases and 20 patients with cirrhosis whose samples were analyzed using the UHPLC-Q-Exactive-MS. Liver cirrhosis had been diagnosed in all HCC patients included in this study. The diagnosis of HCC cases was made using well-established criteria for diagnostic imaging and/or histology. The clinical stages of HCC were defined using the tumor-node-metastasis (TNM) classification system. The overall experimental design, including sample preparation, data acquisition, data processing, statistical analysis, and data interpretation, for untargeted metabolomics and lipidomics analysis is depicted in Figure 1.

### 2.3. Serum Sample Preparation

To conduct metabolomics and lipidomics studies, serum samples were prepared according to the previously published studies with a few modifications [10,16]. For the metabolomics study, 150 μL of ice-cold methanol was added to each 50 μL serum samples aliquot using the serum methanol ratio of 1:3. To obtain better protein precipitation, the sample mixture was then vortex-mixed and centrifuged at 21,913× *g* for 10 min 4 °C. A clear supernatant was then transferred to a fresh tube and diluted with water containing internal standards for positive (debrisoquine sulfate; 2 μg/mL) and negative (4-nitrobenzoic acid; 2 μg/mL) modes at a ratio of 2:1 (supernatant: water). After mild vortexing and spinning, 5 L of the sample was subsequently injected into the UHPLC-Q-Exactive-MS system. A quality control (QC) sample was prepared by obtaining identical volumes of serum samples and diluting them with water containing the IS mixture (2 g/mL) using the same ratio as described above. This QC sample was utilized to give a representative “mean” sample encompassing all analytes encountered during the analysis, and to evaluate the instrument’s stability. The QC sample was injected multiple times at the start of each run to condition or equilibrate the system. It was then injected after every 10 samples to assess the stability of the analysis. The QC data were used to investigate the intra-run analytical variability.

To perform the lipidomics study, an aliquot of 50 μL serum was mixed with 25 μL of PC (16:0/18:1)-d31 (4 μg/mL; internal standard for positive mode), 25 μL of arachidonic acid-d8 (4 μg/mL; internal standard for negative mode), and 50 μL of 0.1 M NaCl. To extract lipids, 250 L of ice-cold chloroform/methanol (1:2; *v*/*v*) was added to the serum mixture, which was then vortexed for 1 min, left at room temperature for 1 h, and followed by centrifugation at 21,913× *g* for 10 min at 4 °C. The organic layer was separated to a new tube and evaporated to dryness using sppedvac. The residue was then reconstituted with 100 μL of ice-cold isopropanol:acetonitrile:water (2:1:1; *v*/*v*) and injected into the instrument for analysis. A QC sample was also prepared by taking equal volumes from each sample after reconstitution in order to assess the instrument’s consistency and reliability. All QC samples were processed in a manner similar to the metabolomics analysis.

### 2.4. Instrumental Conditions

LC-MS/MS data were acquired in both positive and negative modes using a Vanquish UHPLC system connected to a Q-Exactive mass spectrometer (Thermo Fisher Scientific, San Jose, CA, USA) equipped with a heated electrospray ionization (HESI) source.

In the metabolomics study, an ACQUITY UPLC BEH C18 column (2.1 × 100 mm, 1.7 mm, Waters, Milford, MA, USA) was utilized for the chromatographic separation while maintaining the autosampler and column oven temperature at 4 °C and 50 °C, respectively. The mobile phase consisted of 0.1 formic acid in water (*v*/*v*; mobile phase A) and in methanol (*v*/*v*; mobile phase B), and a flow rate of 0.3 mL/min was used for the elution. The elution gradient was controlled as follows: initial elution was at 100% A for 1 min, then it was reduced to 80% A during the next 4 min, and from 4 min to 10 min, mobile phase A was decreased linearly from 80% to 30%. At 14 min, the flow of mobile phase A was brought down to 0% before being rapidly brought back up to the initial conditions for a 2 min re-equilibration phase.

Chromatographic separations for the lipidomics study were executed on an ACE Excel 2 Super C18 column (2.1 × 100 mm, 1.7 mm, Advanced Chromatography Technologies Ltd., Aberdeen, Scotland, UK), with the autosampler and column oven temperature set to 4 °C and 50 °C, respectively. Then, 10 mM ammonium acetate was contained in either 40% acetonitrile (*v*/*v*, mobile phase A) or acetonitrile: isopropanol (10:90, *v*/*v*, mobile phase B) and was eluted at the same flow rate as the metabolomics analysis. The gradient elution was managed in the following manner: the gradient was started with 60% mobile phase A and kept for 1 min, decreased linearly to 35% A over the course of the next 2.5 min, then decreased again from 35% to 0% A over the course of the next 9 min and held at that level for 0.5 min before returning to the initial gradient state of 60% A to re-equilibrate the condition.

The analysis of serum was conducted under identical mass spectrometric (MS) conditions for both metabolomics and lipidomics studies. UHPLC-Q-Exactive-MS data were acquired at a resolution of 70,000 with a centroid mode scan ranging from *m/z* 66.6 to 1000 for metabolomics and 80 to 1200 for lipidomics. The MS/MS scans were performed using 5 loop counts at a resolution of 17,500 by applying stepped normalized collision energy (step-NCE) of 20, 30, and 45 with an isolation window of 2.0 *m/z*. The automatic gain control (AGC) was set to 1 × 10^6^ and 1 × 10^5^ for full MS and dd-MS^2^, respectively, while the dynamic exclusion was set to 30.0 s. The detailed heated electrospray ionization (HESI) source parameters were as follows: capillary temperature was 320 °C; spray voltage was 4.0 kV for positive and 3.8 kV for negative ion modes; sheath gas flow rate was 46.0 arb in positive and 50.0 arb in negative ion modes; auxiliary gas flow rate was 11.0 arb for positive and 10.0 for negative ion modes; sweep gas flow rate was set to 1.0 arb for both ion modes; and the RF level in the S-lens was 60%. Nitrogen was used for both the sheath gas and the auxiliary gas.

### 2.5. Data Processing and Statistical Analysis

The raw Q-Exactive-MS data were processed by Compound Discoverer 3.1 (Thermo Fisher Scientific, San Diego, CA, USA) to align, detect, and identify the peaks. The processed data were then normalized using the peak area of internal standards. Specifically, debrisoquine sulfate and 4-nitrobenzoic acid were used to normalize the positive and negative metabolomics data, whereas PC(16:/18:1)-d31 and arachidonic acid-d8 were used to normalize the positive and negative lipidomics data. Principal component analysis (PCA) and partial least square discriminant analysis (PLS-DA) were performed to visualize the differences and to evaluate the differential metabolites between HCC and LC groups using Metaboanalyst 5.0 following log transformation and Pareto scaling. To identify significantly altered ions, a two-sample *t*-test was used. The *p*-values were then adjusted using the Benjamini–Hochberg false discovery rate. Metabolites were considered significant based on the *p*-value (<0.05) and FDR adjustment (0.05).

### 2.6. Metabolite Annotation

Significantly altered putative metabolites were annotated based on the *m/z* of the mass adducts ([M + H]^+^, [M + Na]^+^, [M + NH4]^+^, [M − H]^−^, etc.) and the MS/MS of fragments of each *m/z* using various tools, compound databases, and spectral libraries including MetaboQuest, Compound Discoverer, LipidSearch, Human Metabolome Database (HMDB), and METLIN.

### 2.7. Network and Pathway Analyses

Network and pathway analyses were conducted using the Ingenuity Pathway Analysis (IPA) software, utilizing all annotated metabolites identified through metabolomics and lipidomics studies.

### 2.8. Receiver Operating Characteristic (ROC) Curve Analysis

The ROC curve analysis was performed to evaluate the diagnostic power of each individual metabolite candidate identified through our metabolomics and lipidomics studies to diagnose the HCC. We compared the area under the curve (AUC) of each metabolite with the AUC of AFP, the most commonly used marker for HCC diagnosis.

## 3. Results

### 3.1. Untargeted Metabolomics and Lipidomics Analysis of HCC vs. LC

**Figure 2:**
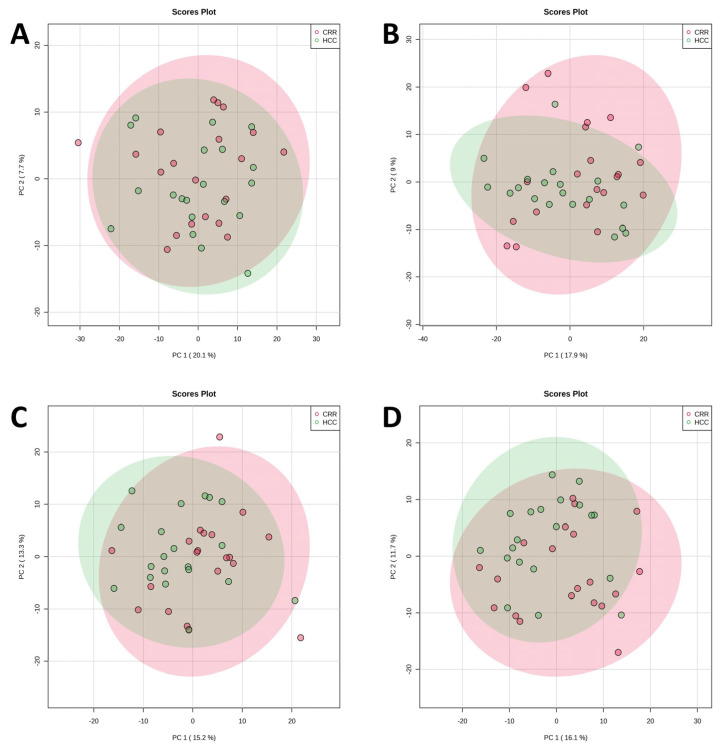
Principal component analysis (PCA) score plot of HCC (green) and cirrhosis (red) cohorts. Metabolomics (**A**) positive and (**B**) negative modes; Lipidomics (**C**) positive and (**D**) negative modes.

Serum metabolomics and lipidomics profiling studies were carried out to identify potential biomarkers associated with HCC development through comparisons with LC. Metabolic and lipidomic features obtained from UHPLC-Q-Exactive-MS analysis were subjected to PCA and PLS-DA analyses to visualize the differences in HCC from LC. The PCA score plots for both the metabolomics and lipidomics analysis in both positive and negative modes showed a partial overlap between HCC and LC groups (Figure 2), which could be because of the similar disease states between these two cohorts. The PLS-DA analysis of the same metabolomics and lipidomics datasets demonstrated a distinct separation between the HCC and LC groups (Supplementary Figure S1) with good modeling and predicting capabilities (R^2^ = 0.99, Q^2^ = −0.04 for metabolomics positive mode, R^2^ = 0.98, Q^2^ = 0.08 for metabolomics negative mode, R^2^ = 0.98, Q^2^ = 0.31 for lipidomics positive mode, and R^2^ = 0.98, Q^2^ = 0.24 for lipidomics negative mode). R^2^ represents the model’s explanation capacity, while Q^2^ denotes its predictive ability; R^2^ and Q^2^ values near 1 indicate that the model is excellent. The overall R^2^ and Q^2^ values demonstrated that the model was reliable and had good predictability. The low Q^2^ value observed in positive metabolomics may be attributed to the partial overlap between the HCC and LC groups. On the contrary, the lipidomics analysis exhibited greater R^2^ and Q^2^ values, indicating a more substantial differentiation between the HCC and LC populations.

**Figure 3:**
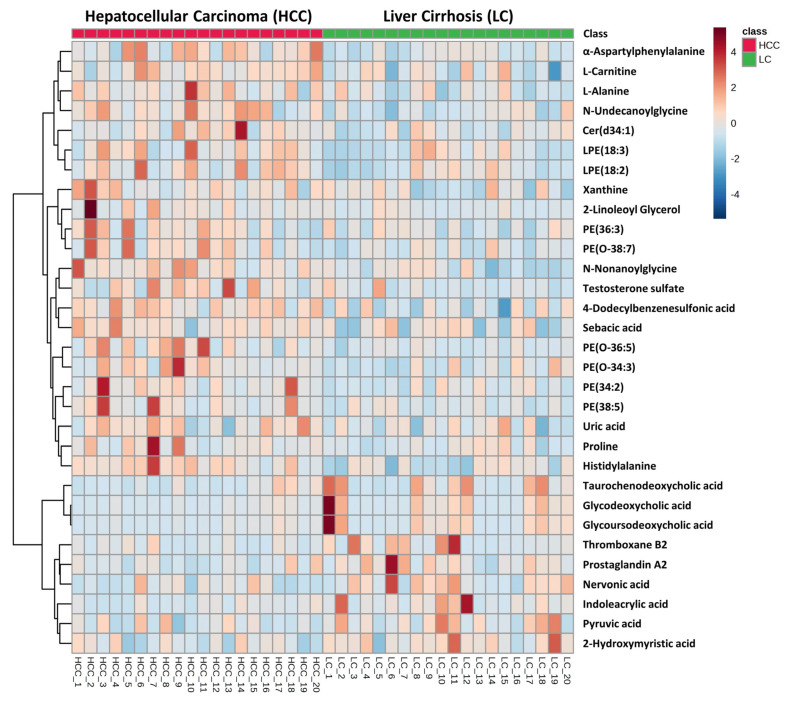
Hierarchical clustering analysis (heatmap) of identified serum metabolites altered in HCC cohort compared to LC cohort in metabolomics study.

In the serum metabolomics study, a total of 20,277 and 9584 analytes were detected by Compound Discoverer 3.1 in positive and negative modes, respectively. Compound Discoverer chose spectra from the original data using a signal-to-noise (S/N) threshold of 3.0. Subsequently, retention time (RT) alignment was performed with an RT tolerance of 0.3 min and a mass precision of 10 ppm. Among detected analytes, the levels of 1017 (in positive) and 559 (in negative) ions were significantly different based on the *t*-test result (*p* < 0.05). Based on *p*-value < 0.05 and fold change ratio > 1.18, 31 putative metabolites of various classes, including carboxylic acid and derivatives, fatty acyls, steroid and steroid derivatives, glycerophospholipids, and a few organic compounds, were identified. Table 2 and Table 3 provides information on the identified serum metabolites in detail. Among these metabolites, bile acids from the steroid and steroid derivatives class displayed the highest differences in the HCC group from the LC group where most of the bile acids were significantly decreased in HCC. All the glycerophospholipids, especially lysophosphatidylethanolamine (LysoPE) and phosphatidylethanolamine (PE), were another class of metabolites that showed significant upregulation in HCC cases. In addition, most of the fatty acids were downregulated in HCC from LC cases and all the metabolites under the class of carboxylic acids and derivatives showed upregulation in the HCC group compared to the LC group. Figure 3 depicts a heatmap of identified metabolites that are differentially expressed based on *p*-values and fold change in HCC vs. LC groups. In addition, Supplementary Figure S2 displays a heatmap with hierarchical clustering performed on both the patient samples and the metabolites. Supplementary Figure S3 displays individual dot plots representing the significantly changed metabolites discovered by the lipidomics analysis between HCC and LC cases. However, despite small *p*-values and high fold change, none of the metabolites passed the FDR cutoff. Thus, targeted quantitation of the selected candidates is highly desired to confirm the observed difference between HCC and LC.

**Figure 4:**
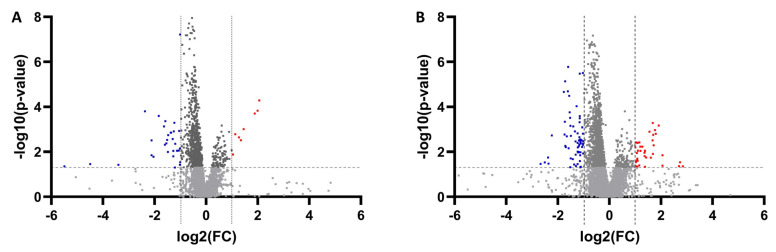
Important features selected by volcano plots with false discovery rate (FDR) < 0.05 and |FC| > 2 from univariate analysis (**A**) positive and (**B**) negative modes. Red-colored dots denote upregulated, blue-colored dots denote downregulated, and gray-colored dots denote metabolites with non-significant change.

**Figure 5:**
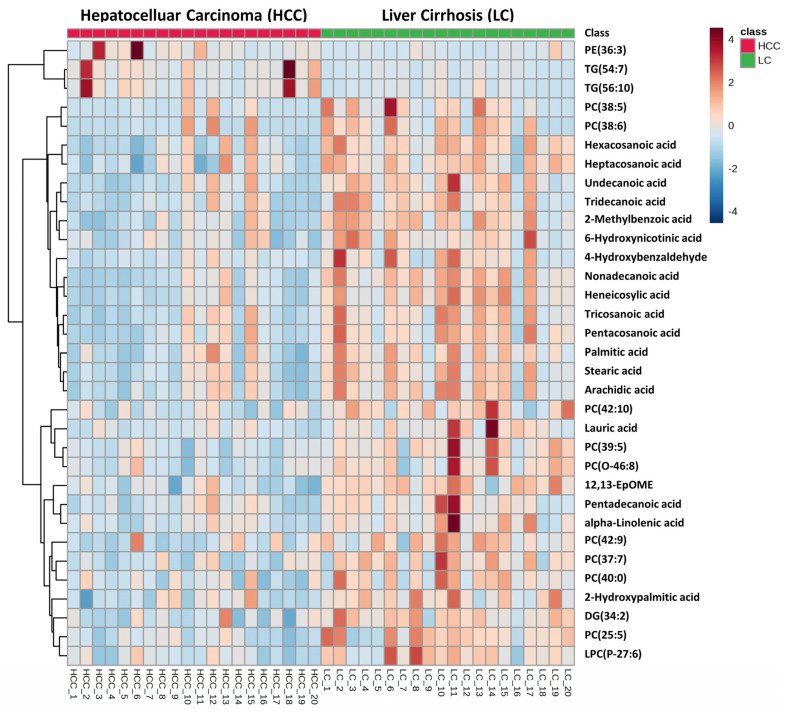
Hierarchical clustering analysis (heatmap) of identified serum metabolites altered in HCC cohort compared to LC cohort in lipidomics study.

For the lipidomics study, a total of 5014 (in positive mode) and 8614 (in negative mode) analytes were detected by Compound Discoverer 3.1 software. After the *p*-value evaluation and adjustment of *p*-value, we found that 276 and 421 metabolites were significantly altered in positive mode and negative mode, respectively. A total number of 33 metabolites were finally annotated by matching their corresponding MS/MS fragments. Figure 4 depicts a volcano plot illustrating the significantly altered metabolites from the univariate analysis. All the annotated metabolites were significantly altered based on both the *p*-value and FDR value in the HCC vs. LC groups. The metabolites belong to the class of fatty acyls, glycerophospholipids, and a few organic compounds. In the lipidomics study, all the fatty acids and phosphatidylcholine (PC) from the glycerophospholipids class were significantly downregulated, whereas the PE and triglyceride were significantly upregulated. Figure 5 depicts a heatmap of the identified metabolites, while Supplementary Figure S4 depicts a heatmap with hierarchical clustering on both the patient samples and the metabolites. Individual dot plots for all of the significantly altered metabolites between HCC and LC cases detected by the lipidomics study are shown in Supplementary Figure S5.

### 3.2. Network and Pathway Analyses

**Figure 6:**
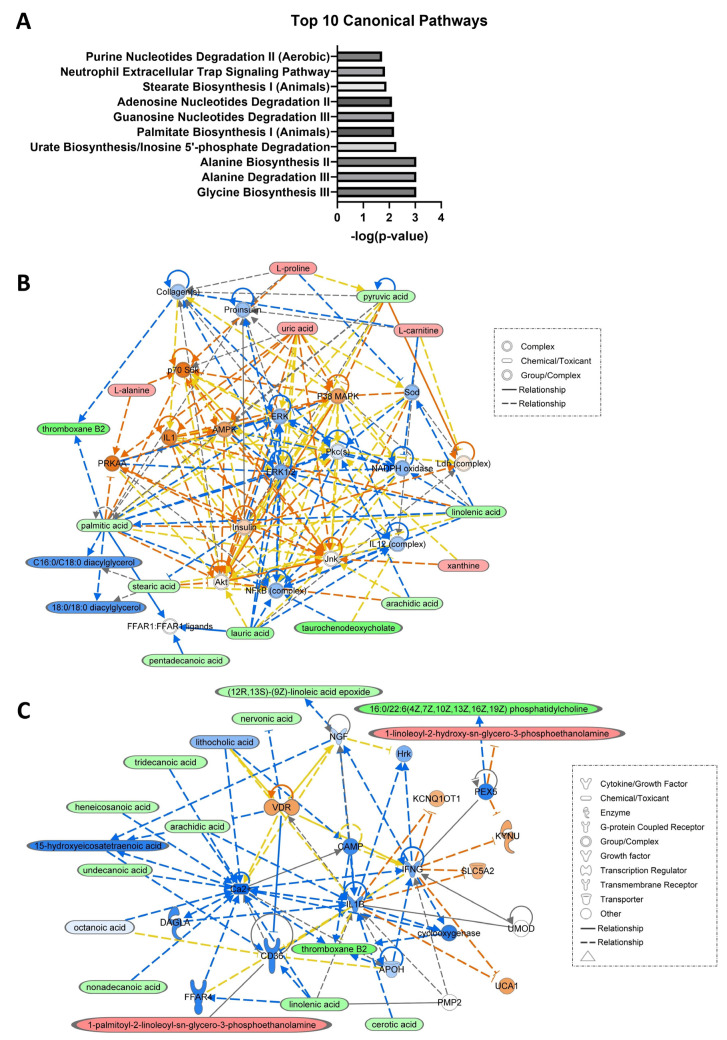
Pathway and network analyses of 51 metabolites mapped by IPA from the metabolite candidates identified by metabolomics and lipidomics studies; (**A**) Top 10 canonical pathways based on 51 mapped metabolites, (**B**) network involving 14 out of the 51 metabolites. (**C**) network involving 13 out of 51 metabolites (upregulated in HCC vs. LC marked in red, downregulated in HCC vs. LC marked in green). Orange line indicates activation, blue line indicates inhibition, yellow line indicates findings inconsistent with state of downstream molecule, gray line indicates effect not predicted, dashed lines indicate indirect relationship, and solid lines indicate direct relationship.

The pathway analysis reveals that the identified biomarker candidates contribute to the enrichment of several canonical pathways, including glycine, alanine, stearate and palmitate biosynthesis, alanine, guanine nucleotides and adenosine nucleotides degradation, urate biosynthesis/inosine 5′-phosphate degradation, and neutrophil extracellular trap signaling pathway. Figure 6A depicts the top 10 canonical pathways identified by IPA based on all metabolites significantly altered in the metabolomics and lipidomics studies. Among the identified biomarker candidates from both the omics studies, IPA utilized a total of 14 metabolites from omics studies that were associated with developmental disorder, hereditary disorder, and metabolic disorder networks. Additionally, IPA found 13 metabolites engaged in cell signaling, molecular transport, and vitamin and mineral metabolism networks. The networks generated through IPA are depicted in Figure 6B,C.

### 3.3. Receiver Operating Characteristic (ROC) Curve Analysis

**Figure 7:**
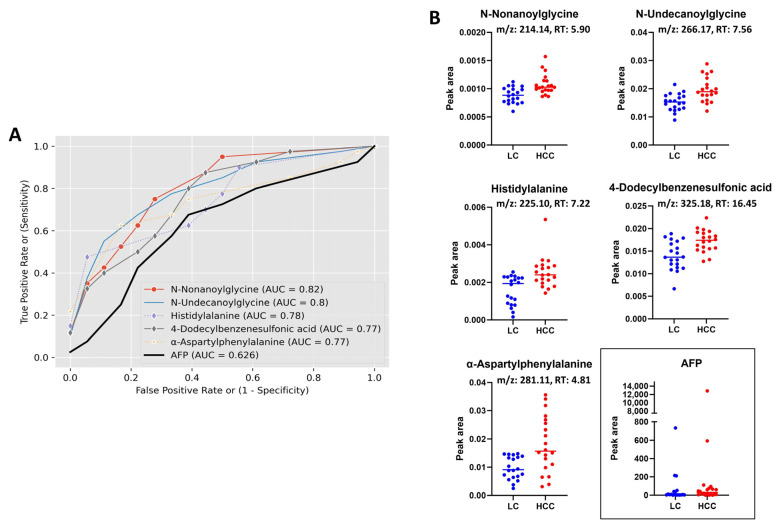
Combined ROC curves and dot plots of top five metabolites having highest AUC value identified by metabolomics study; (**A**) combined ROC curves of top five metabolites including the AFP, (**B**) individual dot plot of corresponding metabolites including the AFP (horizontal lines represent median). RT, retention time.

**Figure 8:**
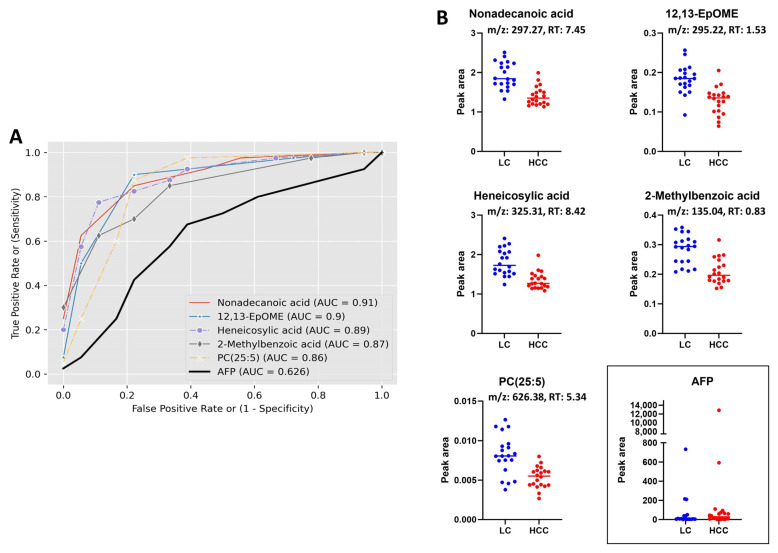
Combined ROC curves and dot plots of top five metabolites having highest AUC value identified by lipidomics study; (**A**) combined ROC curves of top five metabolites including the AFP, (**B**) individual dot plot of corresponding metabolites including the AFP (horizontal lines represent median). RT, retention time.

Based on our data, all the identified biomarker candidates displayed considerably superior performance in comparison to AFP, as evidenced by the area under the receiver operating curve (AUC). According to Xia et al., an area under the curve (AUC) value within the range of 0.9–1.0, 0.8–0.9, 0.7–0.8, 0.6–0.7, and 0.5–0.6 corresponds to the categories of excellent, good, fair, bad, and fail, respectively. These categories are used to assess the predictive capacity of a biomarker in diagnostic applications [17]. The AUC of all our biomarker candidates identified by lipidomics study was >0.71, where the AUC of AFP was 0.62, indicating a clear superiority in performance. Furthermore, the majority of the metabolites identified through the metabolomics study exhibited an AUC of over 0.7, surpassing the performance of AFP as a diagnostic marker. Figure 7A,B and Figure 8A,B present the receiver operating characteristic (ROC) curve and scatter plots, respectively, displaying the top five metabolites with the highest AUC values which were identified using metabolomics and lipidomics investigations. The evaluated area under the receiver operating characteristic curve (AUC) value of each individual biomarker candidate for HCC identified through metabolomics and lipidomics studies is shown in Supplementary Tables S1 and S2.

## 4. Discussion

This study aimed to discover biomarker candidates that can distinguish HCC from LC. To accomplish this, serum samples from 20 HCC cases and 20 patients with LC were analyzed using the untargeted metabolomics and lipidomics approaches. Data acquired utilizing UHPLC-Q-Exactive-MS led to a total of 64 metabolites that were significantly altered in HCC patients than in LC patients. Specifically, we noticed significant differences between the serum samples of HCC patients vs. LC patients at the level of carboxylic acid and derivatives, fatty acyls, steroid and steroid derivatives, glycerophospholipids, glycerolipids, and a few organic acids.

Uric acid (UA) is the final metabolite of purine metabolism in humans, formed from xanthine and hypoxanthine via the action of xanthine oxidase (XOD) and serving dual roles as an antioxidant and prooxidant. The prooxidant role of UA contributes to the production of reactive oxygen species (ROS) that eventually promote tumorigenesis [18]. Previous reports have revealed the paradoxical role of UA due to its upregulation and downregulation of various cancer instances [19]. UA was found to have a positive correlation with colorectal, kidney, nonmelanoma skin, HCC, and other cancers [19]. Recent research found that XOD activity is increased in HCC patients compared to healthy controls [20]. Likewise, another study demonstrated a relationship between increased serum UA and decreased survival in individuals with advanced HCC [21]. In contrary, a negative association of UA was also observed in pulmonary, central nervous system, breast, lymphatic, and other cancers [19]. The results of our metabolomics study of higher levels of xanthine and uric acid in the HCC cohort could be the indicator of higher ROS production which is ultimately influencing the HCC progression.

According to our metabolomics data, in terms of fold change, the highest alteration was found in steroid and steroid derivative metabolites, especially bile acids (BAs). Bile acids are the main constituents of bile and play a pivotal role in multiple biological processes, including the absorption of cholesterol, lipids, and fat-soluble vitamins; regulation of cellular signal; energy metabolism; etc. It has been reported that abnormal levels of BA are associated with liver diseases, particularly HCC [7,22]. In this study, all the bile acids, particularly conjugated bile acids, including glycodeoxycholic acid (GDCA), glycoursodeooxycholic acid (GUDCA), and taurochenodeoxycholic acid (TCDCA), were significantly downregulated in HCC vs. LC. The downregulation of bile acids in HCC compared to LC has been reported previously and also supported by our previous work [23,24,25]. This downregulation of conjugated BA could be the effect of altered bile acid transport pathway in the development of HCC [25].

Since fatty acids (FAs) are the fundamental building blocks of complex lipid species that contribute as an energy source for cells to grow and proliferate, aberrant FA metabolism is a crucial factor for cancer progression, including HCC [26]. With a few notable exceptions, the saturated fatty acids (SFAs) and monounsaturated fatty acids (MUFAs) in our metabolomics data have been greatly reduced. Consistent with our findings, the attenuation of FA levels has been observed previously in numerous cancer studies [27,28]. Patterson et al. reported reduced levels of FAs, particularly nervonic acid, in the plasma of HCC patients compared to the LC patients [29].

Phosphatidylethanolamine (PE) and lysophosphatidylethanolamine (LPE) are the classes of glycerophospholipids (PLs) that showed significant elevation in HCC vs. LC. In mammalian cells, PE is the second most abundant phospholipid. It is abundant in the inner membrane of mitochondria and makes up about 25% of mammalian PLs, both of which are located in the inner leaflet of the plasma membrane [30]. PE plays a role in numerous critical pathologic cellular processes in addition to its role as a membrane structural element [31]. During cell division and cell death, PE is translocated and redistributed to facilitate membrane fusion and remodeling [32]. LPE, on the other hand, is a deacylated product of PE hydrolysis generated by phospholipase A1/A2 and is involved in multiple pathological cellular processes [33]. The dysregulation of PE and LPE levels is frequently observed in a variety of diseases, including cancer [34]. In our metabolomics study, all the PEs and LPEs were significantly upregulated in HCC cases compared to LC, indicating a clear dysregulation of PE and LPE metabolism.

In the lipidomics study, the fatty acids from the fatty acyl class; phosphatidylcholine (PC), lysophosphatidylcholine (LPC), and PE from the glycerophospholipid class; and diglyceride (DG) and TG from the glycerolipids class displayed the most significant alteration in the HCC cases vs. LC patients. Our lipidomics data revealed an even more distinct picture of FA metabolism in HCC compared to LC. We observed a clear, statistically significant difference in the levels of all FAs, and, interestingly, the majority of them were SFAs that exhibited a similar pattern of downregulation observed in metabolomics. The decrease in FA level was also seen in our earlier GC-MS-based metabolomics study [35]. The FA attenuation observed in our data clearly indicates the alteration in FA metabolism in HCC.

PC is the most abundant and fundamental component of the cell membranes. It plays a crucial role in the structure and function of cell membranes and is regarded as one of the hallmarks of cancer growth and progression in a variety of cancer types [10,36]. In our study, the level of all annotated PCs was significantly decreased in HCC cases compared to LC patients. Additionally, only one annotated plasmenyl LPC exhibited the same pattern of downregulation as PC. Phospholipase A2 (PLA2) catalyzes the conversion of PC into lysoPC, a lipid mediator that controls a variety of biological processes, including inflammatory responses, tumor cell invasiveness, and cell proliferation [37]. The downregulation of PCs and LPCs in HCC patients vs. LC is corroborated by multiple prior studies, including ours, and could be the result of decreased hepatocyte functions or the disruption of lipid homeostasis [12,25,38]. In our lipidomics study, we found only one PE (PE(36:3)) which was significantly altered in HCC cases following the same trend of alteration as our metabolomics study. The elevation of PE and LPE and reduction of PC and LPC levels in our metabolomics and lipidomics studies could be due to the reduced expression of PE N-methyltransferase 2 (PEMT2) enzyme which catalyzes the conversion of PE to PC in the liver when dietary choline supply is low and found to be reduced or missing in HCC [25,38]. However, additional study is required to establish a direct correlation between PEMT2 and the observed rise in PE and LPE, as well as the decrease in PC and LPC levels in hepatocellular carcinoma (HCC).

The increased levels of circulatory TGs are a well-known biomarker of liver dysfunction and are reported to have a positive correlation with multiple liver diseases, including cholestasis, ALD, NAFLD, HBV, and HCC [39]. Higher levels of TG are also found in numerous other cancers like gallbladder, cervical, colon, and respiratory cancers [40], while a few studies have reported a negative correlation of TG with prostate and breast cancers [41,42]. According to Liu et al., TG upregulation was observed in HCC patients who had no cirrhosis [43]. In our study, we found a significant elevation in two TG (TG54:7 and TG56:10) levels with a fold change of 4.17 and 3.70 in HCC patients with cirrhosis compared to cirrhotic controls. It is speculated that oxidative stress and reactive oxygen species (ROS) could be the possible factors that contribute to the elevation of TG since these factors are often increased in cancer cells [44]. However, additional molecular-level studies are required to demonstrate the precise mechanism of triglycerides in HCC development.

Based on the findings of the network analysis, it was observed that two prominent networks, depicted in Figure 7A,B, were implicated in developmental disorder, hereditary disorder, and metabolic disease, as well as cell signaling, molecular transport, and vitamin and mineral metabolisms. These networks were constructed using a total of 14 and 13 metabolites, respectively. The networks presented in this study demonstrate the direct and indirect participation of many proteins, transcription factors, receptors, and enzymes, such as AKT, AMPK, P70 S6K, ERK, and CD36, in association with the detected metabolites. Numerous studies have already established the direct and indirect connections between these proteins, transcription factors, receptors, and enzymes and the development, suppression, and progression of various malignancies, particularly HCC [45,46,47,48]. This network analysis provides more evidence of the association between the detected metabolites and hepatocellular carcinoma (HCC), and it suggests that they could be evaluated as potential candidates for biomarkers of HCC.

## 5. Conclusions

In this study, serum metabolomics and lipidomics approaches were used to identify biomarker candidates that can distinguish early-stage HCC from LC. Using the UHPLC-Q-Exactive-MS system, we identified a broad class of metabolites, including organic acids, purine metabolites, fatty acids, bile acids, and lipids, that displayed significant variation in HCC vs. LC. Among these classes, bile acids, fatty acids, and lipids exhibited the greatest variation in HCC. Since 95% (19 out of 20) of our recruited HCC patients were from stages I and II (considered early stages) and were cirrhotic, candidate biomarkers identified in this study will be of interest for the early detection of HCC in patients with LC. To accomplish this, large-cohort studies with independent validation are needed. Thus, future work will focus on confirming the identities of candidate metabolite biomarkers discovered in this study, followed by targeted quantitation in serum samples from an independent cohort with a greater number of participants.

## Figures and Tables

**Table 1:** Clinical characteristics of study population.

Variables		HCC (n = 20)	LC (n = 20)	*p*-Value
Age	Mean (SD)	59 (6)	58 (6)	0.4870
Gender	Male	12	13	1.0000
Race	AA	10	7	0.5231
	EA	10	13	
BMI	Mean (SD)	30 (7.2)	30 (4.8)	0.9663
Etiology	HCV	17	16	1.0000
	Alcohol	6	7	1.0000
HCV Serology	HCV Ab+	16	15	0.6948
HBV Serology	anti HBC+	9	7	0.7475
	HBs Ag+	1	0	1.0000
Smoking	Current	5	5	1.0000
	Former	11	10	
	None	4	5	
Alcohol	Current	5	4	1.0000
	Former	11	12	
	None	4	4	
AFP	Median (IQR)	29.1 (60.8)	7.3 (35.4)	0.3389
AST	Median (IQR)	107.5 (83.2)	94 (70)	0.1903
ALT	Median (IQR)	98.5 (53.2)	53.2 (47)	0.0695
MELD	Median (IQR)	10.5 (5.2)	13.5 (9.3)	0.0474
Child Pugh score	Mean (SD)	6.8 (1.8)	9.1 (2.8)	0.0116
	Median (IQR)	6 (2.5)	9 (5)	
Child Pugh Class	A	9	3	
	B	7	8	
	C	3	5	
HCC Stage	Stage I	6	None	
	Stage II	13		
	Stage III	1		

**Table 2:** List of significantly altered serum metabolites in HCC patients compared to LC patients identified by metabolomics analysis.

Metabolite Name	Class	ID	Exact Mass	*m/z*	RT	Adduct	HCC vs. LC		Fold Change (HCC/LC)	Trend
							*p*-Value	FDR		
Pyruvic acid	Keto acids and derivatives	HMDB0000243	88.0148	87.0070	0.91	[M − H]^−^	0.04081	0.70955	−1.29	↓
L-Alanine	Carboxylic acids and derivatives	HMDB0000161	89.0475	90.0547	0.82	[M + H]^+^	0.00740	0.88660	1.27	↑
Proline	Carboxylic acids and derivatives	HMDB0251528	115.0631	116.0703	1.42	[M + H]^+^	0.04101	0.97773	1.41	↑
N-Undecanoylglycine	Carboxylic acids and derivatives	HMDB0013286	243.1828	266.1720	7.56	[M + Na]^+^	0.00022	0.37484	1.30	↑
N-Nonanoylglycine	Carboxylic acids and derivatives	HMDB0013279	215.1522	214.1444	5.90	[M − H]^−^	0.00062	0.50963	1.22	↑
Histidylalanine	Carboxylic acids and derivatives	HMDB0028878	226.1166	225.1088	7.22	[M − H]^−^	0.00064	0.50963	1.60	↑
α-Aspartylphenylalanine	Carboxylic acids and derivatives	HMDB0000706	280.1052	281.1125	4.81	[M + H]^+^	0.00073	0.49771	1.85	↑
L-Carnitine	Organonitrogen compounds	HMDB0000062	161.1048	162.1120	0.82	[M + H]^+^	0.00813	0.88660	1.24	↑
4-Dodecylbenzenesulfonic acid	Benzene and substituted derivatives	HMDB0251568	326.1919	325.1841	16.45	[M – H]^−^	0.00071	0.50963	1.23	↑
Xanthine	Imidazopyrimidines	HMDB0000292	152.0324	151.0246	1.19	[M − H]^−^	0.02981	0.70955	1.25	↑
Uric acid	Imidazopyrimidines	HMDB0000289	168.0280	169.0353	0.89	[M + H]^+^	0.04447	0.98349	1.32	↑
Indoleacrylic acid	Indoles and derivatives	HMDB0000734	187.0630	188.0703	6.36	[M + H]^+^	0.01981	0.90379	−2.22	↓
Sebacic acid	Fatty acyls	HMDB0000792	202.1200	201.1122	7.06	[M − H]^−^	0.03118	0.70955	1.34	↑
2-Hydroxymyristic acid	Fatty acyls	HMDB0002261	244.2039	243.1961	11.32	[M − H]^−^	0.01980	0.70955	−1.18	↓
Prostaglandin A2	Fatty acyls	HMDB0002752	334.2128	333.2050	10.17	[M − H]^−^	0.03417	0.70955	−1.44	↓
Nervonic acid	Fatty acyls	HMDB0002368	366.3502	365.3424	15.99	[M − H]^−^	0.02158	0.70955	−1.35	↓
Thromboxane B2	Fatty acyls	HMDB0003252	370.2361	369.2283	7.67	[M − H]^−^	0.03649	0.70955	−2.51	↓
2-Linoleoyl Glycerol	Fatty Acyls	HMDB0245187	354.2760	355.2833	12.52	[M + H]^+^	0.04812	0.99111	2.59	↑
Glycodeoxycholic acid (GDCA)	Steroids and steroid derivatives	HMDB0000631	449.3141	432.3098	8.01	[M + H − H_2_O]^+^	0.01730	0.90379	−3.97	↓
Glycoursodeoxycholic acid (GUDCA)	Steroids and steroid derivatives	HMDB0000708	449.3149	448.3071	8.02	[M − H]^−^	0.02246	0.70955	−3.79	↓
Taurochenodeoxycholic acid (TCDCA)	Steroids and steroid derivatives	HMDB0000951	499.2978	498.2900	8.11	[M − H]^−^	0.02940	0.70955	−2.40	↓
Testosterone sulfate	Steroids and steroid derivatives	HMDB0002833	368.1661	367.1583	7.72	[M − H]^−^	0.02959	0.70955	1.85	↑
LysoPE(18:3/0:0); LPE(18:3)	Glycerophospholipids	HMDB0011509	475.2707	474.2629	10.94	[M − H]^−^	0.02921	0.70955	1.62	↑
LysoPE(18:2/0:0); LPE(18:2)	Glycerophospholipids	HMDB0011507	477.2843	478.2916	11.88	[M + H]^+^	0.00765	0.88660	1.62	↑
PE(16:0/18:2); PE(34:2)	Glycerophospholipids	HMDB0008928	715.5165	714.5087	16.67	[M − H]^−^	0.01653	0.70955	1.66	↑
PE(18:1/18:2); PE(36:3)	Glycerophospholipids	HMDB0009027	741.5324	740.5246	16.89	[M − H]^−^	0.04691	0.70955	1.38	↑
PE(18:1/20:4); PE(38:5)	Glycerophospholipids	HMDB0009036	765.5323	764.5245	16.65	[M − H]^−^	0.01554	0.70955	1.60	↑
PE(O−16:1/20:4); PE(O-36:5)	Glycerophospholipids		723.5220	722.5142	16.55	[M − H]^−^	0.00356	0.62660	1.80	↑
PE(O-16:1/22:6); PE(O-38:7)	Glycerophospholipids		747.5222	746.5144	16.45	[M − H]^−^	0.01257	0.70955	1.68	↑
PE(O-16:1/18:2); PE(O-34:3)	Glycerophospholipids		699.5218	698.5140	17.04	[M − H]^−^	0.01861	0.70955	1.49	↑
Cer(d18:1/16:0); Cer(d34:1)	Sphingolipids		583.5179	582.5101	16.44	[M + HCOO]	0.03797	0.70955	1.50	↑

↑: upregulated in HCC, ↓: downregulated in HCC.

**Table 3:** List of significantly altered serum metabolites in HCC patients compared to LC patients identified by lipidomics analysis.

Metabolite Name	Class	ID	Exact Mass	*m/z*	RT	Adduct	HCC vs. LC		Fold Change (HCC/LC)	Trend
							*p*-Value	FDR		
Undecanoic acid	Fatty acyls	HMDB0000947	186.1609	185.1531	2.17	[M − H]^−^	0.00009	0.00700	−1.46	↓
Lauric acid/Dodecanoic acid	Fatty acyls	HMDB0000638	200.1767	199.1689	2.88	[M − H]^−^	0.00034	0.01528	−1.79	↓
Tridecanoic acid	Fatty acyls	HMDB0000910	214.1925	213.1847	3.70	[M − H]^−^	0.00010	0.00724	−1.44	↓
Pentadecanoic acid	Fatty acyls	HMDB0000826	242.2241	241.2163	5.10	[M − H]^−^	0.00239	0.04856	−1.35	↓
Palmitic acid	Fatty acyls	HMDB0000220	256.2398	255.2320	5.65	[M − H]^−^	0.00058	0.02039	−1.18	↓
2-Hydroxypalmitic acid	Fatty acyls	HMDB0031057	272.2351	271.2273	3.27	[M − H]^−^	0.00076	0.02357	−1.37	↓
alpha-Linolenic acid	Fatty acyls	HMDB0001388	278.2224	277.2146	5.09	[M − H]^−^	0.00096	0.02738	−1.61	↓
Stearic acid	Fatty acyls	HMDB0000827	284.2713	283.2635	6.70	[M − H]^−^	0.00008	0.00643	−1.25	↓
12,13-EpOME	Fatty acyls	HMDB0004702	296.2349	295.2271	1.53	[M − H]^−^	0.00003	0.00311	−1.43	↓
Nonadecanoic acid	Fatty acyls	HMDB0000772	298.2870	297.2792	7.45	[M − H]^−^	0.00000	0.00025	−1.38	↓
Arachidic acid	Fatty acyls	HMDB0002212	312.3028	311.2950	7.71	[M − H]^−^	0.00018	0.01032	−1.26	↓
Heneicosylic acid	Fatty acyls	HMDB0002345	326.3182	325.3104	8.42	[M − H]^−^	0.00000	0.00056	−1.34	↓
Tricosanoic acid	Fatty acyls	HMDB0001160	354.3496	353.3418	9.12	[M − H]^−^	0.00045	0.01783	−1.28	↓
Pentacosanoic acid	Fatty acyls	HMDB0002361	382.3809	381.3731	9.98	[M − H]^−^	0.00010	0.00749	−1.33	↓
Hexacosanoic acid	Fatty acyls	HMDB0002356	396.3964	395.3886	10.38	[M − H]^−^	0.00004	0.00352	−1.32	↓
Heptacosanoic acid	Fatty acyls	HMDB0002063	410.4122	409.4044	10.76	[M − H]^−^	0.00056	0.02001	−1.32	↓
4-Hydroxybenzaldehyde	Organooxygen compounds	HMDB0011718	122.0354	121.0276	0.83	[M − H]^−^	0.00138	0.03373	−1.87	↓
2-Methylbenzoic acid	Benzene and substituted derivatives	HMDB0002340	136.0511	135.0433	0.83	[M − H]^−^	0.00000	0.00085	−1.36	↓
6-Hydroxynicotinic acid	Pyridines and derivatives	HMDB0002658	139.0256	138.0178	0.93	[M − H]^−^	0.00072	0.02321	−1.46	↓
PC(22:5/3:0); PC(25:5)	Glycerophospholipids	NIST598	625.3754	626.3833	5.34	[M + H]^+^	0.00010	0.00525	−1.53	↓
PC(22:5/16:0); PC(38:5)	Glycerophospholipids	HMDB0008692	807.5531	830.5635	7.77	[M + Na]^+^	0.00141	0.03407	−2.56	↓
PC(16:0/22:6); PC(38:6)	Glycerophospholipids	HMDB0007991	805.54666	806.5545	7.16	[M + H]^+^	0.00268	0.04897	−2.45	↓
PC(15:1/24:4); PC(39:5)	Glycerophospholipids	NIST3518	821.6588	822.6667	10.85	[M + H]^+^	0.00024	0.00988	−1.59	↓
PC(15:1/22:6); PC(37:7)	Glycerophospholipids	NIST2714	789.5303	790.5382	8.94	[M + H]^+^	0.00054	0.01819	−1.27	↓
PC(20:3/22:6); PC(42:9)	Glycerophospholipids	HMDB0008387	855.5665	856.5743	7.77	[M + H]^+^	0.00187	0.03991	−1.37	↓
PC(18:0/22:0); PC(40:0)	Glycerophospholipids	HMDB0008051	845.6607	846.6685	10.01	[M + H]^+^	0.00190	0.03991	−1.25	↓
PC(20:4/22:6); PC(42:10)	Glycerophospholipids	HMDB0008485	853.5579	854.5657	8.70	[M + H]^+^	0.00189	0.03991	−1.61	↓
LPC(P-27:6)	Glycerophospholipids		621.4174	622.4252	6.65	[M + H]^+^	0.00022	0.00886	−1.29	↓
PC(O-46:8)	Glycerophospholipids		899.6740	900.6818	10.85	[M + H]^+^	0.00115	0.03006	−1.52	↓
DG(20:1/14:1/0:0); DG(34:2)	Glycerolipids	HMDB0007386	592.5066	599.5241	8.35	[M + Li]^+^	0.00012	0.00552	−1.34	↓
PE(18:3/18:0); PE(36:3)	Glycerophospholipids	HMDB0009156	741.5303	740.5225	8.65	[M − H]^−^	0.00127	0.03220	2.93	↑
TG(16:0/16:1/22:6); TG(54:7)	Glycerolipids	HMDB0044079	876.7207	894.7535	13.15	[M + NH4]^+^	0.00005	0.00321	4.17	↑
TG(18:3/18:2/20:5); TG(56:10)	Glycerolipids	HMDB0053065	898.7010	899.7089	13.15	[M + H]^+^	0.00020	0.00833	3.70	↑

↑: upregulated in HCC, **↓**: downregulated in HCC.

## Data Availability

Not applicable.

## Supplementary Materials

The following supporting information can be downloaded at: https://www.mdpi.com/article/10.3390/metabo13101047/s1, Figure S1: Partial least squares discriminant analysis (PLS-DA) score plot of HCC (green) and cirrhosis (red) cohorts. Metabolomics (A) positive (R2 = 0.99, Q2 = −0.04) and (B) negative (R2 = 0.98, Q2 = 0.08) modes; Lipidomics (C) positive (R2 = 0.98, Q2 = 0.31) and (D) negative (R2 = 0.98, Q2 = 0.24) modes; Figure S2: Hierarchical clustering (both left and top) analysis (heatmap) of identified serum metabolites altered in HCC cohort compared to LC cohort in metabolomics study; Figure S3: Individual dot plots of significantly altered metabolites in HCC vs. LC identified by metabolomics study. Horizontal lines represent the median; Figure S4: Hierarchical clustering (both left and top) analysis (heatmap) of identified serum metabolites altered in HCC cohort compared to LC cohort in lipidomics study. Horizontal lines represent the median; Figure S5: Individual dot plots of significantly altered metabolites in HCC vs. LC identified by lipidomics study. Horizontal lines represent the median. Table S1: Evaluated area under the receiver operating characteristic curve (AUC) of individual biomarker candidates for HCC identified by metabolomics study; Table S2. Evaluated area under the receiver operating characteristic curve (AUC) of individual biomarker candidates for HCC identified by lipidomics study.
