# Supplementary material for: Biomarker Discovery for Hepatocellular Carcinoma in Patients with Liver Cirrhosis Using Untargeted Metabolomics and Lipidomics Studies
Source: Metabolites. 2023 Oct 2;13(10):1047. doi: 10.3390/metabo13101047 (PMC10608999; doi:10.3390/metabo13101047)
Supplement: Supplementary file 1 [file metabolites-13-01047-s001.zip › metabolites-2584982-supplementary.pdf]

Supplementary Materials

Biomarker Discovery for Hepatocellular Carcinoma in Patients with Liver Cirrhosis Using Untargeted Metabolomics and Lipidomics Studies

Md Mamunur Rashid, Rency S. Varghese, Yuansong Ding and Habtom W. Ressom *

Lombardi Comprehensive Cancer Center, Department of Oncology, Georgetown University Medical Center, Georgetown University, Washington, DC 20057, USA; mr1785@georgetown.edu (M.M.R.); rsv4@georgetown.edu (R.S.V.); yd239@georgetown.edu (Y.D.)

* Correspondence: hwr@georgetown.edu

Supplementary Table S1. Evaluated area under the receiver operating characteristic curve (AUC) of individual biomarker candidates for HCC identified by metabolomics study.

**Metabolites**	**AUC**
N-Nonanoylglycine	0.825
N-Undecanoylglycine	0.800
Histidylalanine	0.778
4-Dodecylbenzenesulfonic acid	0.772
α-Aspartylphenylalanine	0.772
PE(16:0/18:2); PE(34:2)	0.769
PE(O-16:1/18:2); PE(O-34:3)	0.769
L-Carnitine	0.767
L-Alanine	0.764
2-Hydroxymyristic acid	0.761
PE(18:1/20:4); PE(38:5)	0.758
2-Linoleoyl Glycerol	0.756
PE(O-16:1/20:4); PE(O-36:5)	0.744
PE(O-16:1/22:6); PE(O-38:7)	0.736
LysoPE(18:2/0:0); LPE(18:2)	0.736
LysoPE(18:3/0:0); LPE(18:3)	0.728
Prostaglandin A2	0.722
Pyruvic acid	0.717
Testosterone sulfate	0.711
Nervonic acid	0.697
Indoleacrylic acid	0.697
Cer(d18:1/16:0); Cer(d34:1)	0.694
PE(18:1/18:2); PE(36:3)	0.694
Xanthine	0.689
Thromboxane B2	0.672
Proline	0.672
Glycodeoxycholic acid	0.669
Glycoursodeoxycholic acid	0.656
Uric acid	0.656
Sebacic acid	0.653
Taurochenodeoxycholic Acid	0.642
AFP	0.626

Supplementary Table S2. Evaluated area under the receiver operating characteristic curve (AUC) of individual biomarker candidates for HCC identified by lipidomics study.

**Metabolites**	**AUC**
Nonadecanoic acid	0.91
12,13-EpOME	0.90
Heneicosylic acid	0.89
2-Methylbenzoic acid	0.87
PC(22:5/3:0); PC(25:5)	0.86
PC(15:1/24:4); PC(39:5)	0.85
Hexacosanoic acid	0.85
DG(20:1/14:1/0:0); DG(34:2)	0.85
PC(O-46:8)	0.85
TG(16:0/16:1/22:6); TG(54:7)	0.84
Stearic acid	0.84
Tridecanoic acid	0.84
Heptacosanoic acid	0.83
Undecanoic acid	0.83
Lauric acid/Dodecanoic acid	0.83
Pentacosanoic acid	0.83
Tricosanoic acid	0.81
Palmitic acid	0.81
Arachidic acid	0.81
alpha-Linolenic acid	0.81
LPC(P-27:6)	0.81
PE(18:3/18:0); PE(36:3)	0.81
PC(22:5/16:0); PC(38:5)	0.81
PC(20:4/22:6); PC(42:10)	0.80
4-Hydroxybenzaldehyde	0.80
Pentadecanoic acid	0.79
6-Hydroxynicotinic acid	0.79
TG(18:3/18:2/20:5); TG(56:10)	0.79
2-Hydroxypalmitic acid	0.79
PC(15:1/22:6); PC(37:7)	0.78
PC(20:3/22:6); PC(42:9)	0.77
PC(18:0/22:0); PC(40:0)	0.77
PC(16:0/22:6); PC(38:6)	0.71
AFP	0.63

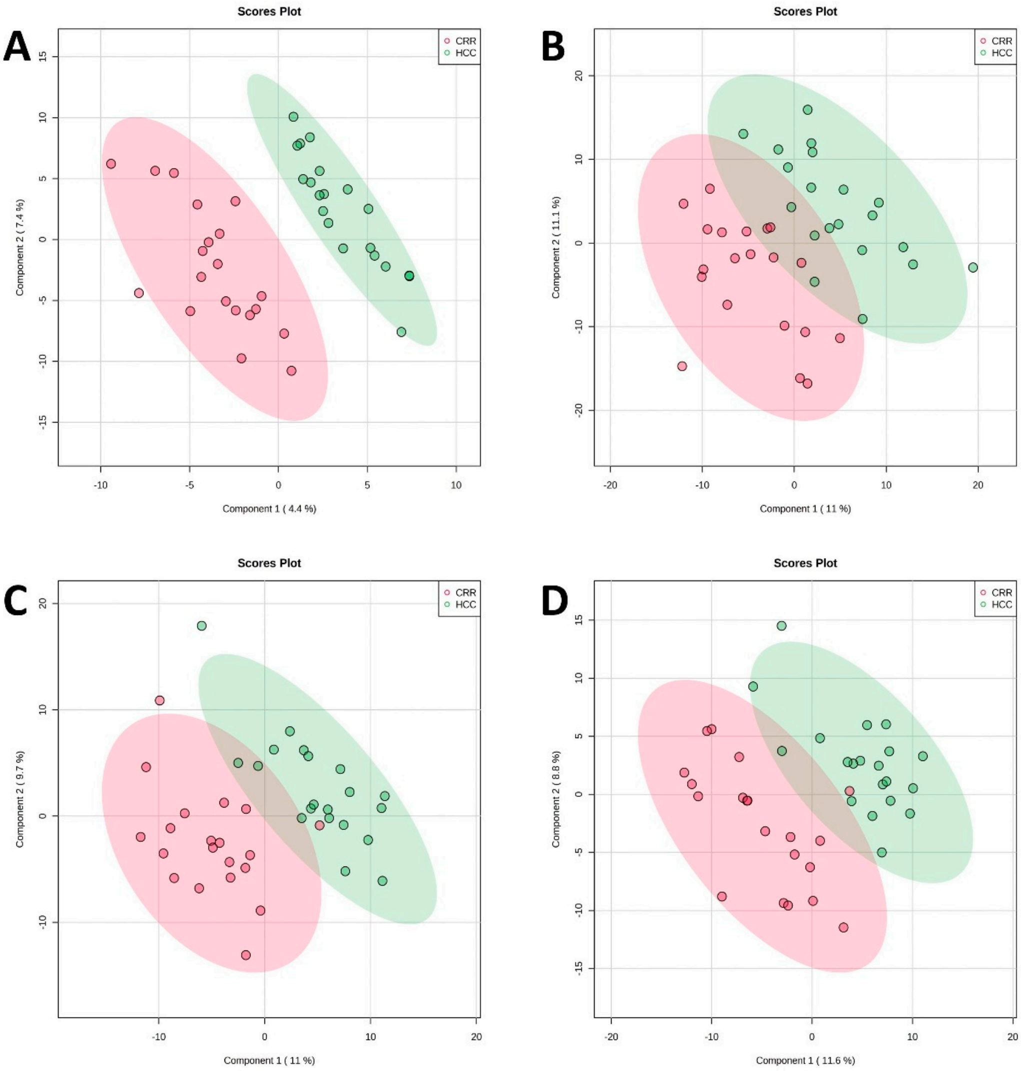

Supplementary Figure S1. Partial least squares discriminant analysis (PLS-DA) score plot of HCC (green) and cirrhosis (red) cohorts. Metabolomics (A) positive (R^2 = 0.99, Q^2 = -0.04) and (B) negative (R^2 = 0.98, Q^2 = 0.08) modes; Lipidomics (C) positive (R^2 = 0.98, Q^2 = 0.31) and (D) negative (R^2 = 0.98, Q^2 = 0.24) modes.

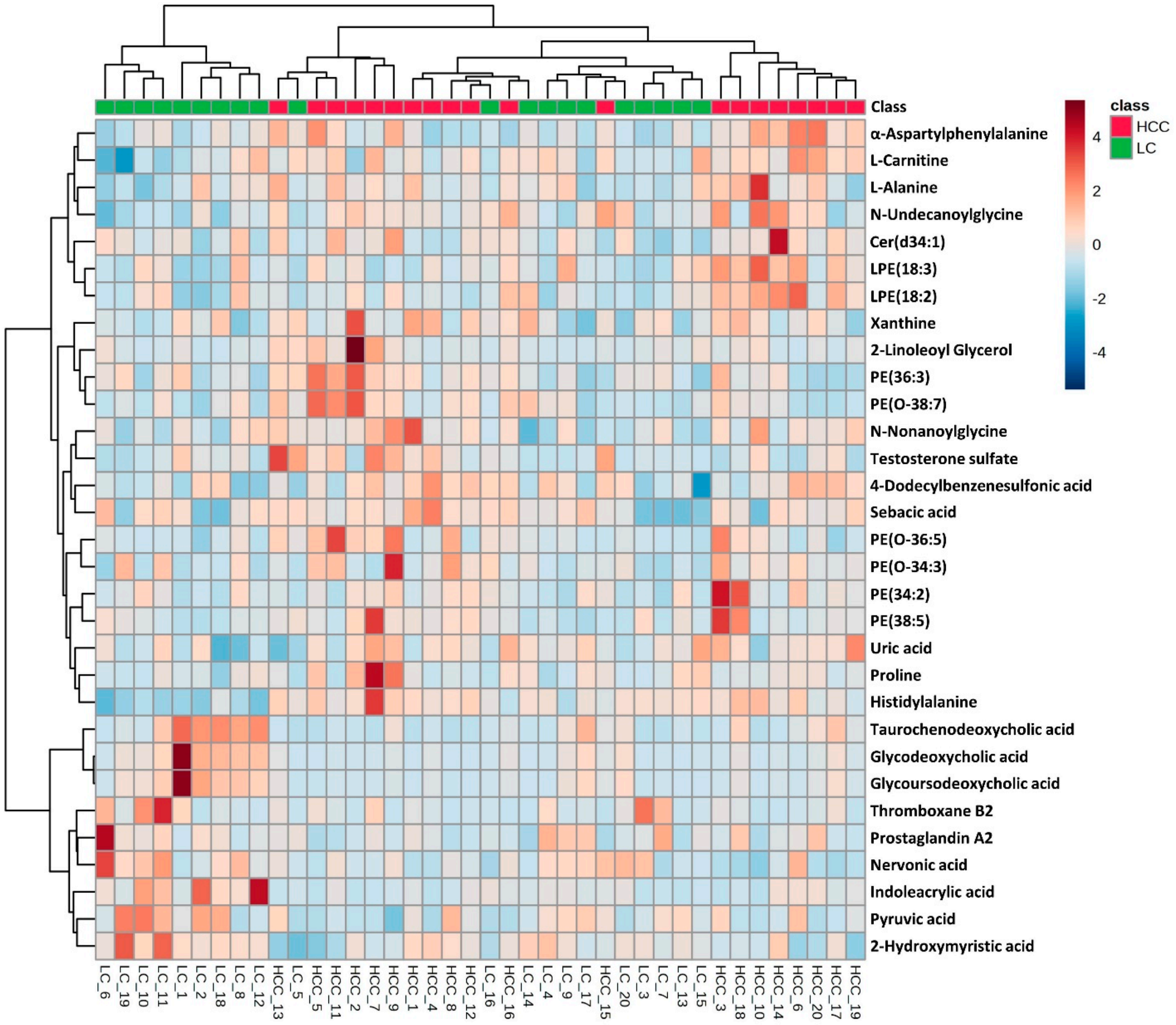

Supplementary Figure S2. Hierarchical clustering (both left and top) analysis (heatmap) of identified serum metabolites altered in HCC cohort compared to LC cohort in metabolomics study.

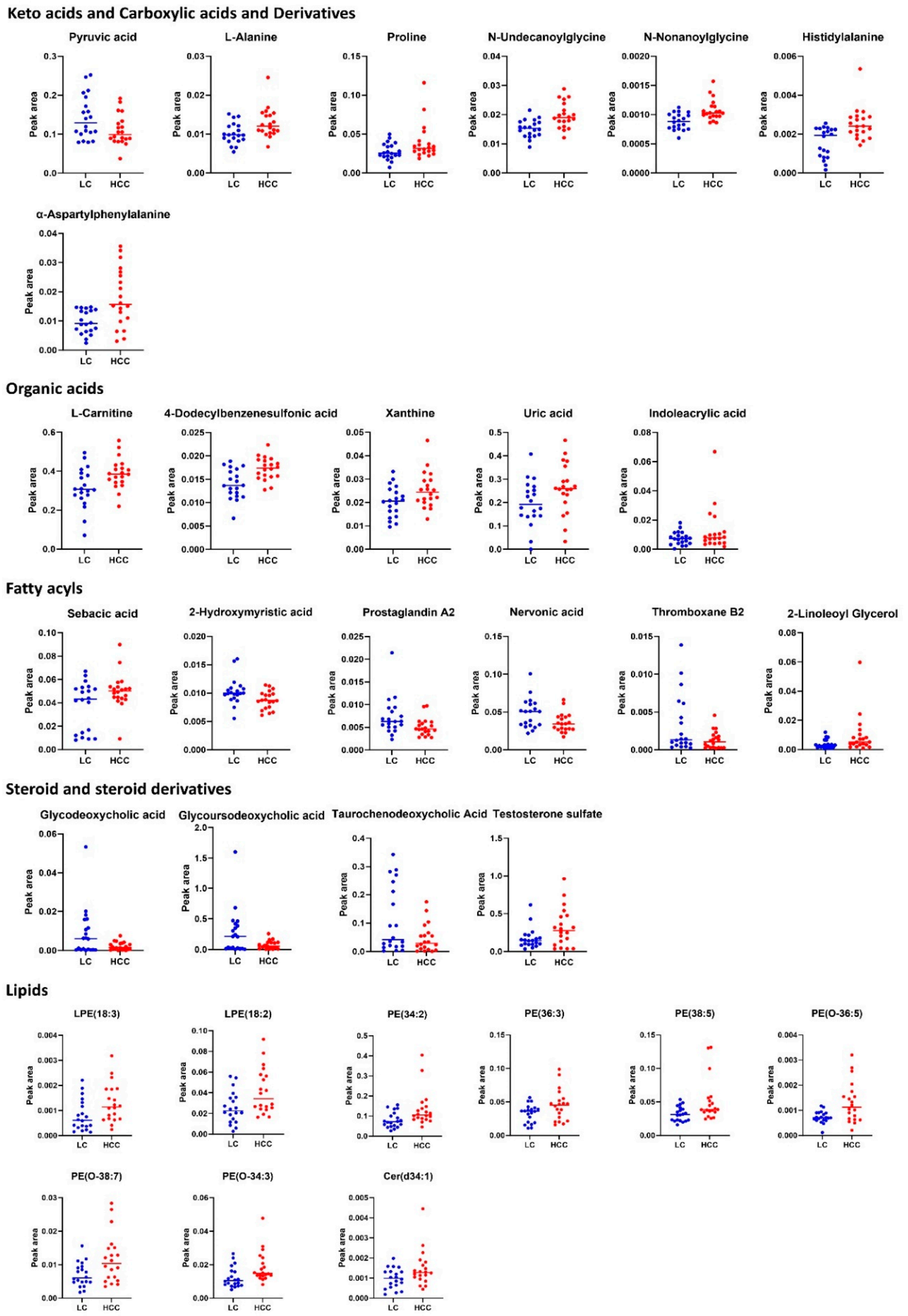

Supplementary Figure S3. Individual dot plots of significantly altered metabolites in HCC vs. LC identified by metabolomics study. Horizontal lines represent the median.

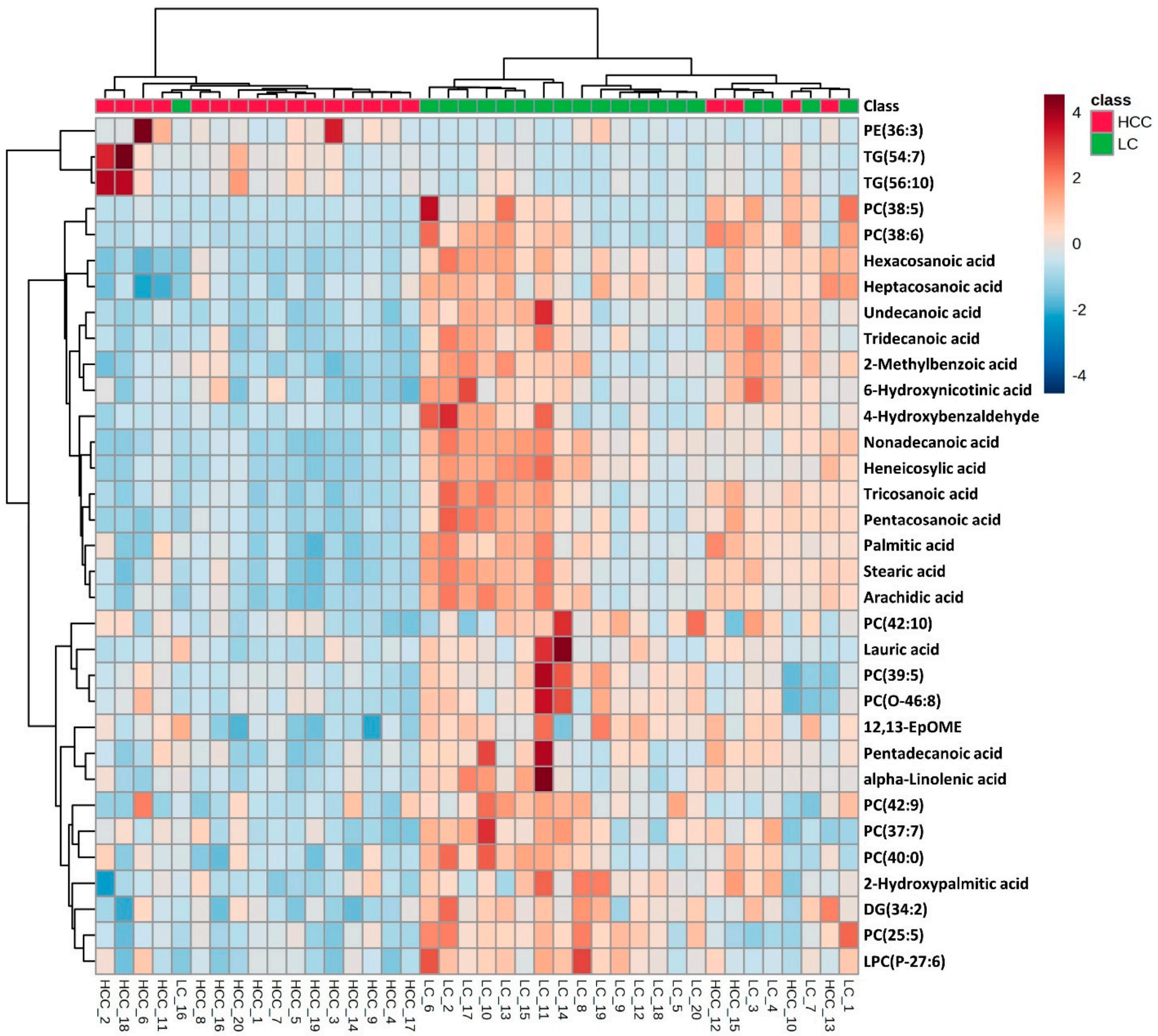

Supplementary Figure S4. Hierarchical clustering (both left and top) analysis (heatmap) of identified serum metabolites altered in HCC cohort compared to LC cohort in lipidomics study.

Fatty acyls

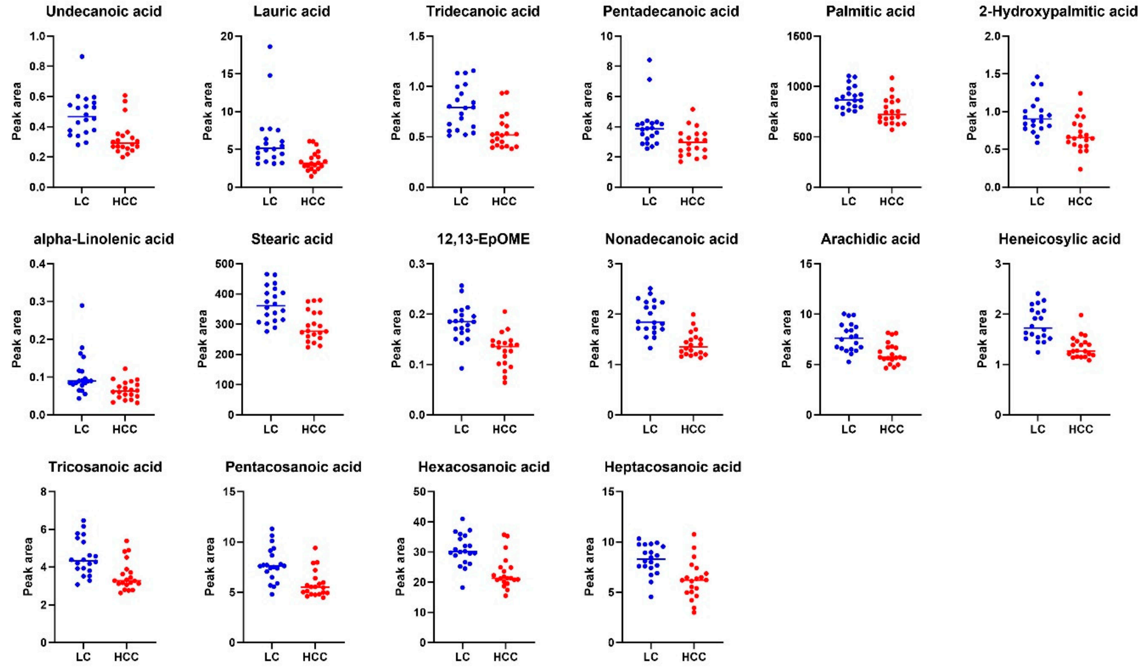

Organic acids

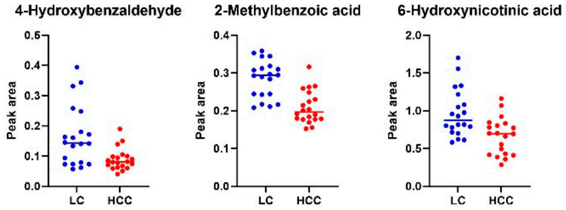

Lipids

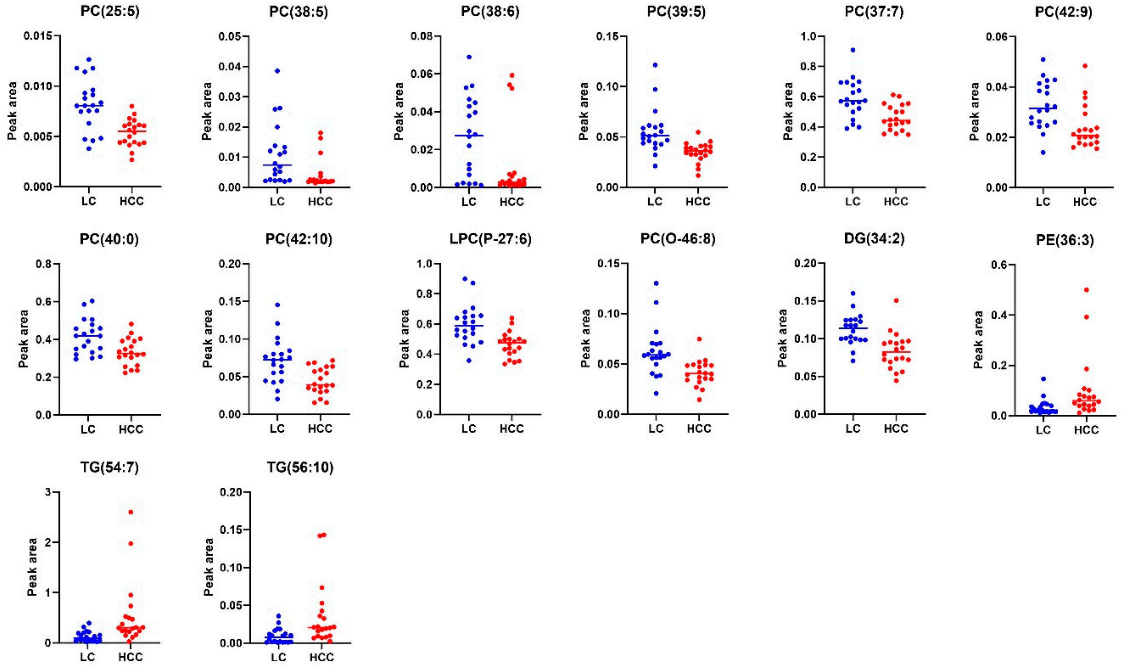

Supplementary Figure S5. Individual dot plots of significantly altered metabolites in HCC vs. LC identified by lipidomics study. Horizontal lines represent the median.
